# A model organism pipeline provides insight into the clinical heterogeneity of *TARS1* loss-of-function variants

**DOI:** 10.1016/j.xhgg.2024.100324

**Published:** 2024-07-02

**Authors:** Rebecca Meyer-Schuman, Allison R. Cale, Jennifer A. Pierluissi, Kira E. Jonatzke, Young N. Park, Guy M. Lenk, Stephanie N. Oprescu, Marina A. Grachtchouk, Andrzej A. Dlugosz, Asim A. Beg, Miriam H. Meisler, Anthony Antonellis

**Affiliations:** 1 Department of Human Genetics, University of Michigan, Ann Arbor, MI, USA; 2 Department of Dermatology, University of Michigan, Ann Arbor, MI, USA; 3 Rogel Cancer Center, University of Michigan, Ann Arbor, MI, USA; 4 Department of Cell and Developmental Biology, University of Michigan, Ann Arbor, MI, USA; 5 Neuroscience Graduate Program, University of Michigan, Ann Arbor, MI, USA; 6 Department of Neurology, University of Michigan, Ann Arbor, MI, USA

**Keywords:** aminoacyl-tRNA synthetases, threonyl-tRNA synthetase, Mendelian disease, recessive disease, protein translation, threonine

## Abstract

Aminoacyl-tRNA synthetases (ARSs) are ubiquitously expressed, essential enzymes that complete the first step of protein translation: ligation of amino acids to cognate tRNAs. Genes encoding ARSs have been implicated in myriad dominant and recessive phenotypes, the latter often affecting multiple tissues but with frequent involvement of the central and peripheral nervous systems, liver, and lungs. Threonyl-tRNA synthetase (*TARS1*) encodes the enzyme that ligates threonine to tRNA^THR^ in the cytoplasm. To date, *TARS1* variants have been implicated in a recessive brittle hair phenotype. To better understand *TARS1*-related recessive phenotypes, we engineered three *TARS1* missense variants at conserved residues and studied these variants in *Saccharomyces cerevisiae* and *Caenorhabditis elegans* models. This revealed two loss-of-function variants, including one hypomorphic allele (R433H). We next used R433H to study the effects of partial loss of *TARS1* function in a compound heterozygous mouse model (R432H/null). This model presents with phenotypes reminiscent of patients with *TARS1* variants and with distinct lung and skin defects. This study expands the potential clinical heterogeneity of *TARS1*-related recessive disease, which should guide future clinical and genetic evaluations of patient populations.

## Teaser

This study leverages an engineered, hypomorphic allele of threonyl-tRNA synthetase (*TARS1*) to capture *TARS1*-associated recessive phenotypes. This strategy revealed both known and previously unappreciated phenotypes, expanding the clinical heterogeneity associated with *TARS1* and informing future genetic and clinical evaluations of patient populations.

## Introduction

Aminoacyl-tRNA synthetases (ARSs) are a family of ubiquitously expressed, essential enzymes that charge tRNA molecules with cognate amino acids, which constitutes the first step of protein translation.^1^ The human nuclear genome encodes 37 ARS loci, with 17 encoding mitochondria-specific enzymes, 18 encoding cytoplasm-specific enzymes, and two encoding enzymes that function in both compartments.^2^^,^^3^ Variants in genes encoding ARSs have been implicated in a spectrum of genetic diseases with all 37 loci implicated in recessive multisystem disorders.^4^^,^^5^^,^^6^ These disorders are caused by bi-allelic variants that severely impair gene function but do not eliminate it, as total loss of any ARS is incompatible with life. Bi-allelic pathogenic variants that affect mitochondrial ARSs tend to cause phenotypes in tissues with a high metabolic demand, including leukoencephalopathies,^7^^,^^8^ myopathies,^9^ and liver disease.^10^^,^^11^ Bi-allelic pathogenic variants in ARS genes encoding cytoplasmic enzymes often affect a wider array of tissues but typically include a neurological component. The recessive neurological phenotypes associated with cytoplasmic ARSs include hypomyelination,^12^^,^^13^ microcephaly,^14^^,^^15^ seizures,^16^^,^^17^ sensorineural hearing loss,^18^^,^^19^ and developmental delay.^20^^,^^21^^,^^22^ Interestingly, variants in some ARS loci cause tissue-restricted or tissue-predominant recessive phenotypes.^23^ For example, although variants in *FARSA*,^24^ *FARSB*,^25^^,^^26^ *IARS1*,^22^ *MARS1*,^27^ and *YARS1*^28^ frequently cause liver dysfunction as one component of a multisystem disease, *LARS1* variants are the most consistent cause of a severe, acute form of infantile liver failure.^29^^,^^30^^,^^31^ Similarly, pulmonary disease is pronounced in individuals with bi-allelic *FARSB*^25^^,^^26^^,^^32^ and *MARS1* variants,^27^^,^^33^ including a *MARS1*-specific form of pulmonary alveolar proteinosis.^34^ The clinical and mechanistic heterogeneities of recessive ARS-related diseases are poorly defined; advancing our knowledge in this area will require generating and characterizing relevant animal models.^35^

Due to the conservation of ARS genes across evolutionarily diverse species, multiple model organisms can be used to study ARS biology and to investigate the impact of pathogenic variants. These models include yeast, worms, fruit flies, and zebrafish.^36^^,^^37^ Mammalian models have historically been limited to studying forms of ARS-mediated dominant peripheral neuropathy^38^^,^^39^^,^^40^ but are increasingly employed for modeling ARS-mediated recessive diseases, including models for *Dars1*,^41^^,^^42^ *Iars1*^43^*, Fars2*,^44^ *Sars2*,^45^ and *Wars2*.^46^ Moving forward, mouse models will be critical tools to understand why certain tissues are particularly sensitive to loss-of-function variants in specific ARS genes, as these questions must be addressed in a model organism with relevant tissue types.

To build a relevant model system pipeline of an understudied ARS gene, we focused on threonyl-tRNA synthetase (*TARS1*). When this study began, *TARS1* had not been implicated in any human disease phenotype. Bi-allelic loss-of-function *TARS1* variants have since been reported in two patients with a recessive brittle hair phenotype.^47^ Additionally, bi-allelic *TARS1* variants were identified in an individual with cerebral palsy and developmental delay, although these variants have not yet been functionally evaluated.^48^ To obtain a more complete assessment of *TARS1*-related recessive phenotypes in a manner that is not limited by patient ascertainment, we generated a model organism pipeline comprising yeast, worm, and mouse. We first engineered three *TARS1* missense variants predicted to cause a loss-of-function effect and tested for these effects in yeast and worm models, which revealed one of these variants (R433H) as a hypomorphic allele. We then used R433H to study the effects of partial loss of *TARS1* function in a compound heterozygous mouse model (R432H/null; R432 is the orthologous position of R433 in mouse *Tars1*). This model presents with some phenotypes that are reminiscent of the trichothiodystrophy recently attributed to bi-allelic *TARS1* variants.^47^ This model also presents with distinct lung and skin phenotypes not previously associated with *TARS1*. In sum, this study expands the potential clinical heterogeneity of *TARS1*-related recessive disease, which should guide future clinical and genetic evaluations of relevant patient populations.

## Results

### Identification of three loss-of-function *TARS1* variants

The model organism *Saccharomyces cerevisiae*, or Baker’s yeast, provides a tractable eukaryotic system for studying highly conserved human genes, as well as disease-associated variants that affect the function of these genes.^49^^,^^50^ For aminoacyl-tRNA synthetase (ARS) genes, *S. cerevisiae* has been a reliable model to study pathogenic variants and test them for loss-of-function effects.^37^ Because ARS genes are highly conserved across evolution, the human open reading frame can often complement loss of the endogenous *S. cerevisiae* ortholog, resulting in a “humanized” yeast model. In this model, yeast growth is interpreted as a proxy for ARS function, since loss of ARS function will impair cell survival and colony formation.

**Figure 1:**
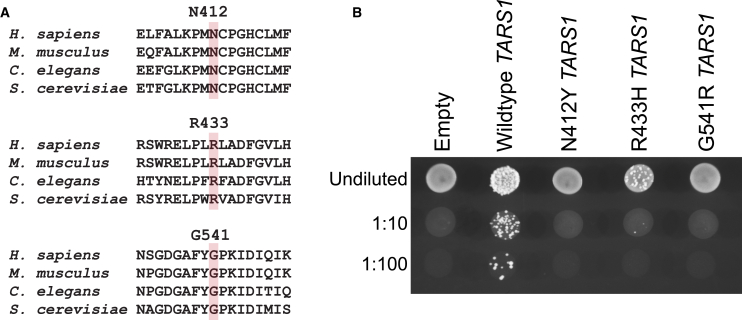
Engineered *TARS1* variants display a loss-of-function effect in *S. cerevisiae* (A) Conservation analysis of N412, R433, and G541 *TARS1* in *H. sapiens* (NP_001245366.1), *Mus musculus* (AAH55371.1), *C. elegans* (NP_001022033), and *S. cerevisiae* (NP_116578.3). The targeted residues are highlighted in pink, surrounded by flanking sequences from evolutionarily diverse species. (B) A representative image is shown from three replicates of *S. cerevisiae* haploid strains with *THS1* deleted and transformed with a vector with no *TARS1* insert (“Empty”), or with one to express wild-type, N412Y, R433H, or G541R *TARS1.* Yeast samples were spotted on media containing 5-FOA in serial dilutions (undiluted, 1:10, or 1:100) and then grown at 30°C.

To design candidate recessive variants in *TARS1*, we mutated three residues in the *TARS1* open reading frame that are highly conserved among human, mouse, worm, and yeast (Figure 1A). These variants—GenBank: NP_001245366.1:p.(N412Y), GenBank: NP_001245366.1:p.(R433H), and GenBank: NP_001245366.1:p.(G541R)—were designed to recapitulate the types of amino-acid changes frequently observed in patient populations. These variants were also designed in the aminoacylation domain of the protein to increase the chance that they would disrupt the protein’s catalytic function. To assess whether these variants affect human *TARS1* function, a complementation assay was performed using an *S. cerevisiae* strain with the endogenous *THS1* deleted. Yeast viability was maintained with a pRS316 vector^51^ expressing the yeast *THS1*, along with *URA3.* The pYY1 vector^52^ expressing either wild-type or mutant human *TARS1* was transformed into yeast, then yeast were plated on 5-FOA, which selects for the loss of the maintenance vector expressing *URA3* and *THS1*.^53^ Wild-type *TARS1* supported yeast growth, demonstrating that human *TARS1* can function in yeast (Figure 1B). Transformation with N412Y *TARS1* or G541R *TARS1* did not lead to formation of colonies, indicating that these two variants significantly impair *TARS1* function. Transformation with R433H *TARS1* did support some yeast growth but caused significantly reduced colony formation compared to wild-type *TARS1* (Figure 1B), indicating partial impairment of *TARS1* function (*i.e.*, a hypomorphic allele).

### Homozygosity for G540R *tars-1* is lethal in worm

**Figure 2:**
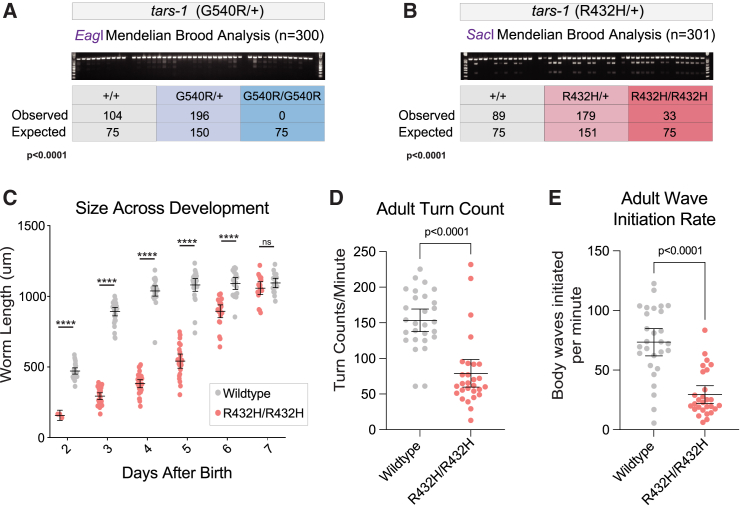
R432H *tars-1* impairs viability and locomotion and delays development in *C. elegans* (A) Genotype analysis of offspring from five broods of G540R/+ *tars-1* hermaphrodites. A representative genotyping gel image is shown. The observed number of each genotype is shown, along with the expected number from Mendelian segregation of a benign variant. A chi-square test between observed and expected numbers was performed to determine statistical significance (*p* < 0.0001). (B) Genotype analysis of offspring from four broods of R432H/+ hermaphrodites, exactly as described in (A). (C) Measurements of body length of R432H/R432H *tars-1* worms and wild-type *tars-1* worms at 6 days after birth. On day 2, *n* = 3 for R432H/R432H, then *n* = 18–30 worms for each subsequent day. For wild-type worms, *n* = 18–30 for each day. (D) Turn count per minute for R432H/R432H worms (*n* = 28) and wild-type worms (*n* = 28) at adult stage P9. (E) Number of body waves initiated from either the head or the tail per minute, for R432H/R432H worms (*n* = 28) and wild-type worms (*n* = 28) at P9. For (C)–(E), bars indicate the mean value and 95% confidence intervals. Statistical significance was evaluated using an unpaired t-test with Welch’s correction; ∗∗∗∗*p* < 0.000001; ns = not significant.

To explore how loss-of-function *TARS1* variants impact the physiology of a multicellular organism, we modeled two of the above variants—the severe loss-of-function G541R and the partial loss-of-function R433H—in worm (*Caenorhabditis elegans*). These variants were each introduced into the endogenous worm *tars-1* locus using CRISPR-Cas9-mediated gene editing, with synonymous variants introduced in *cis* to create restriction digest sites to facilitate genotyping (*EagI* for G540R and *SacI* for R432H) (Figure S1). Of note, the worm amino acid number differs from the human number by one (Table S1). To minimize effects from possible off-target CRISPR variants, G540R/+ worms were back-crossed to the ancestral N2 strain five times, and R432H/+ worms were back-crossed six times. Then, to assess if either of these variants were grossly deleterious in the homozygous state, heterozygous hermaphrodites were allowed to self-fertilize, and offspring were genotyped at the late larval L4 stage or early P1 adult stage to detect deviation from expected Mendelian ratios. In the case of the G540R/+ hermaphrodites, no G540R/G540R offspring were recovered out of 300 worms, indicating that homozygosity for G540R is lethal (Figure 2A). These data confirm that G540R is a loss-of-function allele, validating the results of the yeast complementation assay (Figure 1B). However, because the G540R/G540R genotype did not produce viable animals for additional phenotypic characterization, this variant was not included in further studies. In contrast, when the offspring of R432H/+ hermaphrodites were genotyped, R432H/R432H homozygotes were identified. Only 33 homozygotes were identified, whereas 75 would be expected if homozygosity for R432H was benign (*p* < 0.0001; Figure 2B). This indicates that homozygosity for R432H is deleterious but not completely lethal, consistent with the yeast complementation data indicating that R432H is a hypomorphic allele (Figure 1B).

### R432H *tars-1* causes recessive developmental delay and locomotion defects in worm

One possible explanation for the depletion of R432H/R432H *tars-1* worms in the Mendelian analysis was that this population was under-sampled compared with R432H/+ and wild-type worms. This might occur if developmental delay prevented them from reaching the genotyping time point at the same rate as wild-type or R432H/+ *tars-1* worms. To investigate this possibility, a population of R432H/R432H *tars-1* worms and wild-type N2 worms were age-synchronized. The physical size of the cohort was tracked for over 7 days. Beginning 48 h after hatching, worm length was measured each day using the WormLab video and software system. R432H/R432H *tars-1* worms were consistently smaller than wild-type controls until day 6 or 7 (Figures 2C and S2A). Whereas wild-type worms reach a mature size of approximately 1 mm 3–4 days after birth, R432H/R432H *tars-1* worms do not reach this size until 6–7 days after birth.

We next investigated whether R432H might impair locomotion. We performed a thrash assay with adult worms 9 days after they reached adulthood (P9). Here, R432H/R432H *tars-1* worms were age-matched to wild-type N2s by synchronizing embryo production. The WormLab video capture and analysis system was used to record 1-min videos of worms swimming in M9 buffer, track their motion, and calculate locomotion parameters. R432H/R432H worms display significant thrash impairment (Figures 2D and 2E; Video S1), indicating that reduced *tars-1* function affects the neuronal circuitry or muscular function governing worm locomotion. This locomotion defect was also present in the L4 larval stage, although less pronounced (Figures S2B and S2C). Combined with the significant delay in body size, these data indicate that R432H *tars-1* produces significant phenotypes in a multicellular eukaryotic organism.

Video S1. R432H/R432H worms display significant locomotion defects A 10-s video of adult (P9) wild-type worms (N2 strain) thrashing in liquid M9 media, followed by a 10-s video clip of P9 R432H/R432H *tars-1* worms thrashing in liquid M9.

### Partial loss of *Tars1* function causes neonatal lethality in mouse due to lung defects

To determine how R433H impacts a more complex mammalian system—including defining any specific tissues that might be especially sensitive to partial loss of *TARS1* function—we developed a mouse model of R433H *TARS1.* The R432H variant was introduced into the mouse *Tars1* locus using CRISPR-Cas9-mediated gene editing. (Like *C. elegans*, the mouse amino acid number is 432 [Table S1].) To first determine if R432H caused neonatal lethality in the homozygous state, a Mendelian analysis was performed on the offspring of a *Tars1*^R432H/+^ heterozygote intercross. Out of 43 genotyped offspring, R432H homozygotes were recovered at a frequency that did not significantly deviate from the predicted 25% (Figure S3A) and were grossly normal throughout adulthood. These observations are consistent with our data from yeast and worm (see above) demonstrating that R432H *Tars1* retains partial function. However, it also suggests that, unlike yeast and worm, mice are less sensitive to this degree of *Tars1* impairment, and that additional reduction of *Tars1* function might be needed to observe a phenotype in a mammalian model. To this end, we generated a mouse *Tars1* null allele (F538Kfs∗4) and crossed mice heterozygous for this lesion to *Tars1*^R432H/R432H^ homozygous mice. The F538Kfs∗4 null allele produces a premature stop codon in exon 14, leading to a reduction in protein levels (Figure S3C) and homozygous lethality (S3B). The R432H/F538Kfs∗4 genotype resembles many individuals with recessive ARS-mediated disease who are compound heterozygous for a hypomorphic missense allele and a null allele.^4^

**Figure 3:**
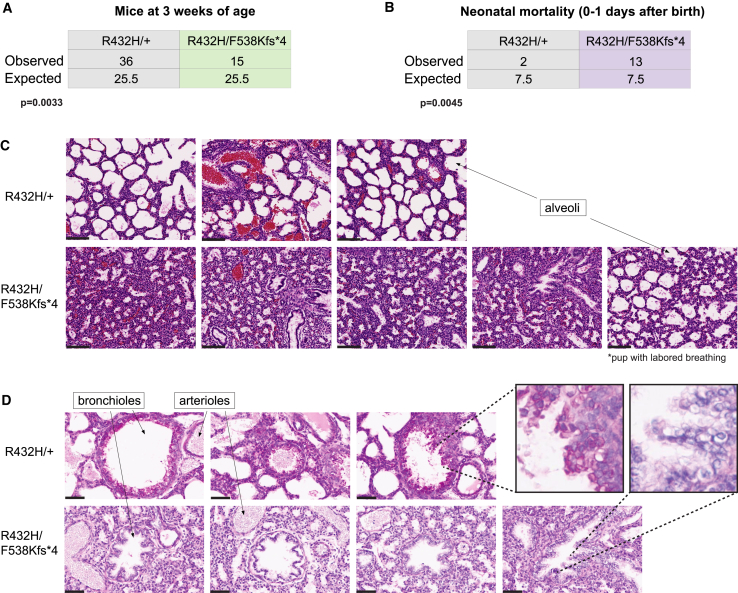
Depleted *TARS1* function causes reduced viability and a lung phenotype in a mouse model (A) Genotype analysis of *Tars1*^R432H/R432H^ and *Tars1*^F538Kfs∗4/+^ offspring, genotyped upon weaning at 3 weeks of age. The observed and expected number of each genotype is shown. (B) Genotype analysis of 15 deceased pups, identified within 1 day after birth. The observed and expected number of each genotype is shown. For (A) and (B), a chi-square test was used to determine if the difference between the number of observed and expected genotypes was statistically significant. (C) H&E staining of lung sections from three *Tars1*^R432H/+^ P0 pups (top row) and five *Tars1*^R432H/F538Kfs∗4^ P0 pups (bottom row). All *Tars1*^R432H/+^ pups were alive when identified at P0. The first four *Tars1*^R432H/F538Kfs∗4^ pups were dead at P0; the fifth was found alive with a gasping, labored breathing pattern. Arrows point to examples of alveoli, which are collapsed in R432H/+ mice. The black scale bar is 100 μm. (D) PAS staining of lung sections from three *Tars1*^R432H/+^ P0 pups (top row) and four *Tars1*^R432H/F538Kfs∗4^ P0 pups (bottom row), with labeled examples of bronchioles and arterioles. The inset highlights the magenta PAS signal in the bronchioles of *Tars1*^R432H/+^ mice, and the absence of PAS signal in the collapsed bronchioles of *Tars1*^R432H/F538Kfs∗4^ mice. The black scale bar is 50 μm.

To assess the effect of the R432H/F538Kfs∗4 *Tars1* genotype on viability, offspring of the cross between *Tars1*^R432H/R432H^ and *Tars1*^F538Kfs∗4/+^ mouse strains were genotyped at 3 weeks of age. Out of 51 pups, we only recovered 15 *Tars1*^R432H/F538Kfs∗4^ (Figure 3A) offspring, indicating decreased viability prior to 3 weeks. An analysis of neonate deaths across four litters showed that pups that died at P0 were enriched for the R432H/F538Kfs∗4 genotype; out of 15 genotyped animals presenting with neonatal death, 13 were R433H/F538Kfs∗4 mice (Figure 3B). To gain insight into the neonatal pathology, a cohort of four P0 *Tars1*^*R432H/F538Kfs∗4*^ pups and three age-matched *Tars1*^*R432H/+*^ littermates were collected for histology. The four *Tars1*^R432H/F538Kfs∗*4*^ pups all died within a few hours of birth. Interestingly, one additional *Tars1*^*R432H/F538Kfs∗4*^ pup was found immediately after birth with traces of birth fluids still visible, exhibiting visibly labored breathing and a failure to right itself. This additional pup was included in the cohort to assess for a respiratory phenotype. All pups were fixed in formalin overnight and washed with 70% ethanol. Subsequently, sagittal sections were prepared and stained with H&E to detect gross morphological changes, and with periodic acid-Schiff (PAS) staining to detect changes in glycoproteins and mucins.

The primary finding from H&E staining was an absence of air in the lungs of the four P0 *Tars1*^R432H/F538Kfs∗*4*^ mice that died shortly after birth. Whereas the alveoli of *Tars1*^R432H/+^ control animals were expanded with air, the alveoli of *Tars1*^R432H/F538Kfs∗^ mice were collapsed (Figure 3C). Considering the otherwise mature body development of these pups, this indicates that they died upon birth or immediately afterward from an inability to breathe. Interestingly, the additional *Tars1*^R432H/F538Kfs∗4^ pup found alive immediately after birth had only partially expanded alveoli, which correlates with the observed labored breathing. Additionally, while the bronchioles of *Tars1*^R432H/+^ control mice are replete with the magenta PAS+ signal of secretory cells, this signal is absent from the collapsed bronchioles of *Tars1*^R432H/F538Kfs∗4^ animals (Figure 3D). To determine if the absent PAS+ signal indicated a loss of these secretory club cells or a significant impairment in their function, we stained similar sections with an antibody against club cell secretory protein (CCSP), an abundant lung protein primarily produced and secreted by the bronchiolar club cells in mouse.^54^ This revealed no significant difference in CCSP levels between *Tars1*^R432H/F538Kfs∗4^ mice and control littermates (Figure S4), indicating that these club cells are present and grossly functional. Another possible explanation for the reduction in bronchiolar PAS+ signal was a reduction of specific threonine-rich glycoproteins like mucins, which may be poorly translated in cells with reduced *Tars1* function. Previous work in pancreatic cancer cells demonstrated that threonine starvation or knockdown of *TARS1* decreases mucin 1 (MUC1) protein levels.^55^ As MUC1 is also a critical airway protein,^56^ we stained our P0 pup lung sections with anti-MUC1 to determine whether decreased MUC1 levels were responsible for the decreased PAS+ signal. There was no significant difference between Muc1 signal in *Tars1*^R432H/F538Kfs∗4^ pups and their littermate controls (Figure S4). Further investigation will be required to determine the underlying mechanism of the loss of PAS+ signal and the pathophysiology of lung dysfunction in mutant *Tars1* mice.

### Partial loss of *Tars1* function causes growth restriction with skin and hair abnormalities

**Figure 4:**
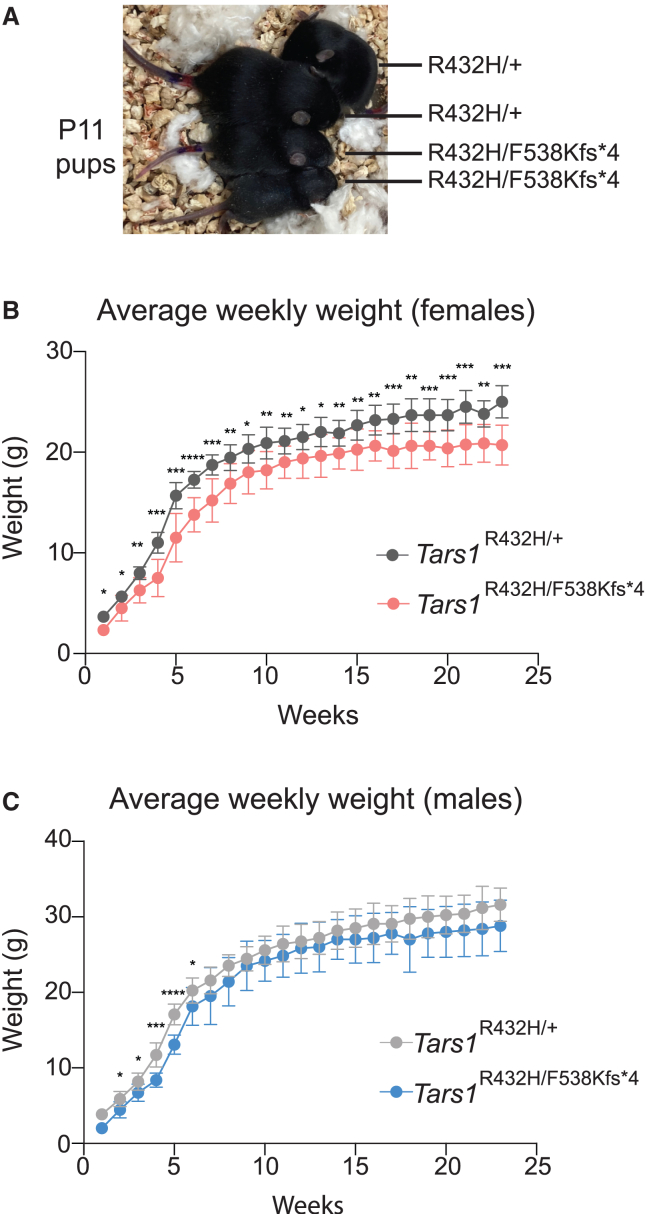
Depleted *Tars1* function causes reduced body size in a mouse model (A) Image of four littermates at P11, grouped together for comparison of body size. The genotype of each mouse is provided. (B) The average weekly weights of female *Tars1*^R432H/F538Kfs∗4^ mice (*n* = 9) and female *Tars1*^R432H/+^ (*n* = 11) littermates are shown, until 23 weeks of age. (C) The average weekly weights of male *Tars1*^R432H/F538Kfs∗4^ mice (*n* = 6) and male *Tars1*^R432H/+^ (*n* = 12) littermates are shown, until 23 weeks of age. For (B) and (C), bars represent the mean value and 1 standard deviation. An unpaired t-test was performed for each week to determine if the difference between the two genotypes was statistically significant. ∗∗∗∗*p* < 0.0001, ∗∗∗*p* < 0.001, ∗∗*p* < 0.01, ∗*p* < 0.05. All values in (C) that are not marked with an asterisk are not significantly different.

While the studies described here were under way, a report of two patients with bi-allelic *TARS1* variants and triochothioydstrophy (TTD) was published.^47^ The phenotypes described in these two patients included delayed physical development, ichthyosis, and collodion baby, and the brittle hair of TTD. Interestingly, *Tars1*^R432H/F538Kfs∗4^ mice display phenotypes that are reminiscent of these human disease features. For example, *Tars1*^R432H/F538Kfs∗4^ mice that survived to adulthood were, on average, smaller than their *Tars1*^R432H/+^ littermates (Figure 4). Reduced body weight was more consistent in females (Figure 4B) than males (Figure 4C), who reach a normal body size by 7 weeks of age. This reduced size is consistent with the delayed physical development described in *TARS1* patients,^47^ and with the growth restriction phenotypes in patients with other ARS-mediated recessive disease.^14^^,^^22^^,^^32^^,^^57^

**Figure 5:**
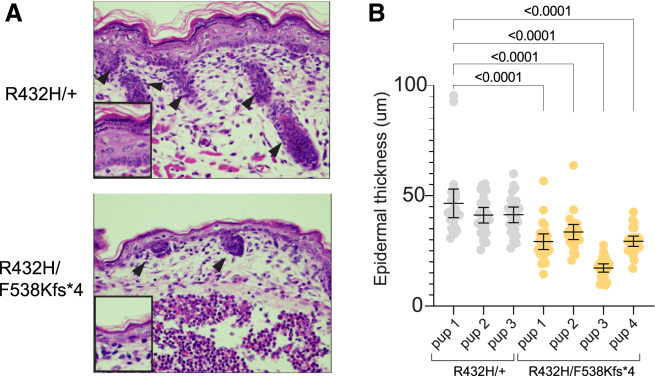
Depleted *Tars1* function causes skin phenotypes in a mouse model (A) H&E staining of dorsal skin sections from P0 pups. The upper panel shows a representative image of skin from a *Tars1*^R432H/+^ mouse, and the bottom panel shows a representative image of skin from a *Tars1*^R432H/F538Kfs∗4^ mouse. Black arrows point to hair follicles in each image. (B) Measurements of epidermal thickness on four *Tars1*^R432H/F538Kfs∗4^ P0 pups and three *Tars1*^R432H/+^ P0 littermates (*n* = 25 measurements per pup). Bars indicate the mean value and 95% confidence interval. Statistical significance was determined with a one-way ANOVA with Šidák’s multiple comparisons testing, comparing all animals with R432H/+ pup 1. Only *p* values < 0.05 are shown (differences between R432H/+ pups were not statistically significant).

**Figure 6:**
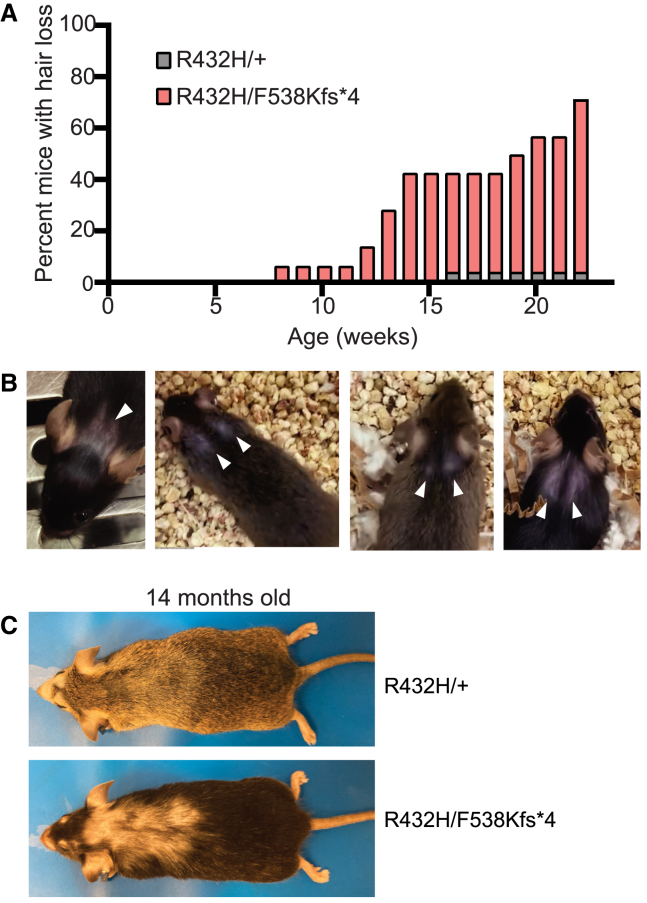
Depletion of *Tars1* function causes hair loss in a mouse model (A) The cumulative percentage of *Tars1*^R432H/F538Kfs∗4^ mice (pink, *n* = 14) and *Tars1*^R432H/+^ mice (gray, *n* = 23) with hair loss on the back of the head or upper back is shown, until 23 weeks of age. (B) Representative images of four individual *Tars1*^R432H/F538Kfs∗4^ mice with hair loss; white arrows point to the consistent pattern of upper back bald patches. The depicted mice are between 10 weeks and 17 weeks of age. (C) A representative image of hair phenotypes in *Tars1*^R432H/+^ (top) and *Tars1*^R432H/F538Kfs∗4^ (bottom) animals. Note that extended hair loss stretches from the head to the middle of the back in the *Tars1*^R432H/F538Kfs∗4^ mouse, at 14 months of age. A *Tars1*^R432H/+^ littermate is shown above, with signs of barbering by the nose and mild age-related hair thinning on the back.

We also detected skin and hair abnormalities in the *Tars*^*R432H/F538Kfs∗4*^ P0 pups and adult mice. The pups had a thinner epidermal layer than control littermates, with fewer layers of stratum corneum (Figures 5A and 5B). They also displayed variable degrees of hair follicle hypoplasia (Figure 5A). This is unlike the only existing mouse model of TTD, which models a patient mutation in *XPD*, the most frequently mutated TTD gene.^58^ In contrast to the *Tars1* mice, this TTD model exhibits a thicker epidermal layer. Adult *Tars1*^R432H/F538Kfs∗4^ mice also displayed a striking postnatal hair phenotype, although it did not resemble the sparse, brittle hair associated with TTD, and was not as severe as the hair loss previously described in the TTD mouse model.^58^ In a nine-litter cohort, 10 out of 14 *Tars1*^R432H/F538Kfs∗4^ mice (71.4%) lost hair on their heads and/or upper back by 23 weeks of age, compared with one out of 23 *Tars1*^R432H/+^ littermates (4.4%). Hair loss onset occurred between 13 and 23 weeks of age (Figure 6A) and followed a stereotypic pattern of bald spots on the head and/or along the scapula of the upper back (Figure 6B). In more advanced stages, it spanned the entire upper back (Figure 6C), although it did not encompass the majority of the body as previously described for TTD mice, nor did it grow back in cycles of loss and regrowth.^58^ To more thoroughly define this phenotype, histopathology was performed on hair samples from the affected regions for three *Tars1*^R432H/F538Kfs∗4^ mice and three *Tars1*^R432H/+^ littermates; one pair was 2 months old, another was 12 months old, and the third was 14 months old. Analysis of H&E staining did not reveal gross abnormalities in hair follicles (data not shown), although this analysis was complicated by the asynchronous hair cycling of adult mice. We also did not observe the classic “tiger tail banding pattern” seen under polarizing microscopy hair from TTD patients.^59^ Taken together, our data demonstrate that partial loss of *TARS1* function causes unusual hair and skin phenotypes in mouse, impairs body weight, and causes a partially penetrant but severe respiratory deficiency.

## Discussion

In this study, we leveraged the established characteristics of pathogenic ARS variants to develop a model system pipeline for predicting the clinical heterogeneity of *TARS1*-related recessive disease. We successfully engineered two loss-of-function *TARS1* missense variants, including a partial loss-of-function allele, R433H, that we employed to explore recessive phenotypes across three model organisms. This analysis revealed that R433H: (1) partially reduced yeast growth in yeast complementation assays; (2) caused developmental delay and locomotion defects in homozygous worms; and (3) caused lung failure, decreased body size, and skin and hair defects when modeled *in trans* with a null allele in mice. While skin and hair phenotypes are seen in our mouse mutants, they do not precisely mimic those recently described for humans with *TARS1* variants. The remaining phenotypes are unique and have not been previously described. While there may be many reasons for this, one possible explanation is that while the well-characterized human missense variants lie within the N2 editing domain, R433H lies within the aminoacylation domain.^47^^,^^60^ This raises the possibility that there are subtle differences in how these variants impact cellular physiology. Overall, our data indicate that phenotypic heterogeneity will ultimately be observed in human *TARS1*-related recessive disease, and that individuals with bi-allelic *TARS1* variants should be carefully evaluated for lung disease.

It is interesting to consider why some tissues may be particularly sensitive to reductions in *TARS1* function. One possibility is that critical proteins with a particularly high threonine content, such as mucins, are more dramatically affected by decreased *Tars1* activity. This could lead to defects in the tissues that rely heavily on these proteins, such as the lung. Interestingly, the gut is also dependent on mucin synthesis.^61^ Although preliminary investigation of gut histology in P0 mice did not identify any changes in PAS signal, careful assessments for gut phenotypes should be performed on patients who are homozygous or compound heterozygous for pathogenic *TARS1* variants. Another possibility is that decreased *Tars1* activity reduces the available population of charged tRNA^Thr^, triggering eIF2α phosphorylation, which then leads to decreased global protein translation. This might affect cells with a high demand for protein translation, such as transient-amplifying progeny of stem cells. For example, if aging hair follicle stem cell progeny cannot properly translate the large mass of proteins required for differentiation of the multiple cell types comprising the mature hair follicle and hair shaft, this could explain a failure to regrow hair in the adult *Tars1*^R432H/F538Kfs∗4^.

In summary, this study demonstrates the efficacy of using variant engineering and a tiered model organism approach to predict the pathogenicity of variants in ARS genes. While additional research on human subjects and animal models will be required to fully define the clinical heterogeneity of *TARS1*-related disease, the data presented here should be useful in genetic and clinical evaluation of relevant patient populations.

## Materials and methods

### Generation of *TARS1* expression constructs

The open reading frame (ORF) of the *TARS1* transcript (GenBank: NM_001258437) was amplified from HeLa cell cDNA, using primers with the attB1 and attB2 gateway recombination sequences (primer sequences in Table S2). These amplicons were purified with Qiagen Spin Miniprep columns and recombined into pDONR221 using Gateway cloning technology (Invitrogen). The recombination reaction was then transformed into Top10 cells (Invitrogen) to isolate clonal populations. Individual bacterial colonies were selected and grown in media containing kanamycin, which selected for the kanamycin resistance cassette on pDONR221. Plasmids were then isolated using the Qiagen Miniprep kit and genotyped by digesting with *Bsr*GI (New England Biolabs) to detect the presence of the *TARS1* insert. Clones with successful insertions were analyzed by Sanger sequencing to ensure absence of variants introduced by amplification errors. To introduce variants into the *TARS1* ORF, site-directed mutagenesis was performed with the QuickChange II XL Site-Directed Mutagenesis Kit (Agilent) (primer sequences Table S2). The reaction was transformed into Top10 cells and grown in Lysogeny broth containing kanamycin to select for pDONR221. Plasmid DNA was isolated and sequenced as above, to ensure successful mutagenesis. Then, the Gateway LR reaction was used to recombine the wild-type or mutant *TARS1* into the vector pYY1. This vector has a 2-μm origin of replication, resulting in a high copy number per cell, as well as the *ADH1* promoter, resulting in strong constitutive *TARS1* expression. Recombinants were transformed into Top10 cells, which were plated on ampicillin to select for the ampicillin resistance cassette on pYY1. Plasmids were extracted, purified, and digested with *Bsr*GI to identify successfully recombined clones.

### Yeast complementation assays

Yeast complementation assays were performed with the Δ*THS1* strain (Horizon Discovery, Clone ID 21471). Yeast viability was maintained with a pRS316 vector that expresses wild-type *THS1* from the endogenous *S. cerevisiae* promoter. pRS316 also carries the auxotrophic marker *URA3* and has a yeast centromere sequence that results in a low copy number per cell. The pYY1 vector (expressing wild-type *TARS1*, mutant *TARS1*, or an empty control) was transformed into yeast with a standard lithium acetate transformation, performed at 30°C with 200 ng of plasmid. Yeasts were grown on solid media without uracil and leucine, which selected for cells with both pRS316 and pYY1. Yeasts were grown for 3 days at 30°C, then individual colonies were picked into 2 mL liquid media lacking uracil and leucine. These cultures were grown for 2 days at 30°C, shaking at 275 rpm. Then, 1 mL of saturated culture was centrifuged at 15,000 rpm for 1 min and cell pellets were re-suspended in 50 μL water. Yeasts were serially diluted to 1:10, 1:100, or 1:1,000 using water. Ten microliters of each dilution (included undiluted yeast) was spotted on complete media containing 5-FOA (Teknova), which selects for cells that have spontaneously lost the pRS315 vector expressing *URA3* and *THS1*.^53^ After 3 to 5 days, yeast growth was visually inspected.

### Generation of the G540R and R432H *tars-1 C. elegans* strains

To generate the G540R and R432H *tars-1* models, CRISPR-Cas9 genome editing was performed according to previously described methods.^62^ Briefly, the gonadal tract of P1 adult worms was injected with an injection mix of: 300 mM KCl, 20 mM HEPES, 2.5 ng/μL pCFJ90, 50 ng/μL single-stranded oligonucleotide homologous donor repair template (Integrated DNA Technologies), 5 μM single guide (sg) RNA (Synthego), and 5 μM Cas9 protein (Integrated DNA Technologies). Sequences for the repair templates and guide RNAs can be found in Table S3. Injected worms were then placed on single 35-mm plates of nematode growth media (NGM) and fresh OP50 bacteria as a food source. Approximately 2 days after injection, plates were screened for the presence of F1 progeny expressing the pCFJ90 marker, which expresses mCherry in the pharyngeal muscles. This enriches for worms that were exposed to the injection mix, increasing the likelihood of identifying a worm subjected to genome editing. The mCherry-positive F1s were singled to individual plates and allowed to produce their own offspring (F2). Then, the F1 worms were placed in lysis buffer (50 mM KCl, 10 mM Tris-HCl pH 8.3, 2.5 mM MgCl_2_, 0.45% NP-40, 0.45% Tween 20, 1 mg/mL proteinase K) and lysed with incubation at −80°C for 1 h, incubation at 65°C for 1 h, and incubation at 95°C for 15 min. To genotype worms, the targeted *tars-1* region was amplified by PCR (primer sequences in Table S2) using Q5 PCR mix (New England Biolabs). Amplicons were then purified with DNA Clean and Concentrator kits (Zymo Research) and digested with the appropriate restriction enzyme (*Eag*I for G540R or *Sac*I for R432H, New England Biolabs). Digested PCR products were separated on a 1% agarose gel and analyzed to identify successful integration of the restriction site. The undigested PCR product from F1s with successful gene editing events was submitted for Sanger sequencing to confirm proper insertion of the restriction site and the desired *tars-1* variant. The offspring of these F1 worms were then maintained for subsequent experiments. To reduce possible off-target variants caused by CRISPR-Cas9 editing, R432H/+ *tars-1* worms were back-crossed to the ancestral N2 strain six times. To assess the Mendelian ratios of the offspring of R432H/+ *tars-1* worms, 6–8 worms from the R432H/+ strain were singled to individual 35-mm plates with OP50, allowed to self-fertilize and produce progeny, then genotyped to confirm heterozygosity for R432H. After confirmation, individual progeny were picked into wells of a 96-well plate for genotyping and Mendelian ratio analysis. This was repeated four times for a total of 301 genotyped offspring.

### Measuring worm body size through development

To identify differences in rates of development, R432H/R432H *tars-1* worms and wild-type N2 worms were first age-synchronized by placing approximately 25 adult worms on a 60-mm plate with NGM and OP50, letting them produce embryos for 4–5 h, and then removing the adults. After 48 h, worms were transferred to unseeded 35-mm NGM plates in batches of 4–5 worms. These worms were filmed and analyzed using the WormLab System (MBF Biosciences). Plates were filmed for 30-s intervals, with the camera set at 4.81 μm/pixel for R432H/R432H worms (Setting 1 on the WormLab camera apparatus [MBF Bioscience]) and 8.47 μm/pixel for N2 worms (Setting 3 on the WormLab camera apparatus [MBF Bioscience]). After filming, worms were moved to new NGM plates seeded with OP50. Filming was repeated every 24 h up to 168 h, or 7 days, after birth (as R432H/R432H worms increased in size, filming was performed with the camera setting at 8.47 μm/pixel). All videos were analyzed with the WormLab software (MBF Bioscience), and the “worm length” parameter was extracted to compare the size of R432H/R432H *tars-1* worms and N2 worms over the course of development.

### Worm thrash assays

Thrash assays were performed to detect changes in worm movement. The bottom of each well of a Nunc 4-well dish (Thermo Scientific) was coated with 2.5% agarose. An amount of 500 μL liquid M9 media (22 mM H_2_KO_4_P, 42 mM HNa_2_O_4_P, 85 mM NaCl, 1 mM MgSO_4_) was added to each well, and 1–5 worms (wild-type or R432H/R432H *tars-1*) were placed in the M9. Worms were allowed to acclimate for 30–60 s before they were filmed with the WormLab System (MBF Biosciences) for 1 min. Only worms with at least 1,000 frames or 30 s of high-quality video were included in subsequent analysis. To identify defects in locomotion, the WormLab parameters “Turn count” and “Wave initiation rate” were analyzed.

### Generation of *Tars1* mouse lines

The R432H variant was introduced into the mouse *Tars1* locus using CRISPR-Cas9-mediated gene editing, which was performed by the University of Michigan Transgenic Animal Core. A single-stranded oligonucleotide (ssODN) was designed to introduce the R432H variant *in cis* with synonymous variants that ablated a *Bgl*I cut site that is present in the wild-type allele, and prevented binding of the guide RNA after repair. Cas9, sgRNA, and ssODN were injected into hybrid C57BL/6J × SJL/J F1 zygotes, which were implanted into pseudopregnant females. These mice produced 32 pups, which were genotyped by PCR amplification (primer sequences in Table S2) and *Bgl*I digestion to identify mice that had incorporated the repair template. Amplicons were submitted for Sanger sequencing to identify mice with proper integration of the repair template. These mice were mated to C57BL/6 mice to establish germline transmission. To assess the Mendelian ratios of offspring genotypes, 43 pups from crosses between *Tars1*^R432H/+^ females and *Tars1*^R432H/+^ males were genotyped using the *Bgl*I restriction enzyme digest strategy described above. Details for generating the F538Kfs∗4 allele can be found in the supplemental information.

### Preparation of mouse tissues for histology

To investigate P0 pups for gross histological changes, dead pups were collected and live pups were killed by decapitation. Pups were individually fixed in neutral-buffered formalin, rocking overnight at room temperature. Pups were then placed in 70% ethanol and stored at 4°C. To investigate histological changes in the hair and skin of adult mice, three *Tars1*^R432H/F538Kfs∗4^ mice and their age-matched, sex-matched *Tars1*^R432H/+^ littermates were euthanized. The mice were shaved, and skin was collected from the dorsal trunk, ventral trunk, ears, tail, and paws, as well as from the area of the head with visible hair loss. Skin samples were placed on 0.45-μm HA filters (Millipore) wetted in PBS and strips were cut parallel to the direction of hair follicle growth. All strips were then fixed overnight in neutral-buffered formalin at room temperature, transferred to 70% ethanol, and stored at 4°C. Samples were shipped to Histoserv, Inc. for embedding and sectioning. Briefly, samples with bone were decalcified, tissues were dehydrated, and water inside of the tissues was replaced with paraffin wax. Tissues were then embedded into wax blocks of paraffin. Blocks were sectioned and affixed to slides (two sagittal sections were taken for the P0 pups). Adult skin sections were stained with H&E; P0 pup sections were stained with either H&E or PAS, which detects glycoproteins and mucins.

### Analysis of epidermal thickness in P0 pups

Dorsal skin from H&E-stained sections was used to analyze the epidermal thickness of four *Tars1*^R432H/F538Kfs∗4^ mice and three *Tars1*^R432H/+^ littermates. Five 1-mm areas were selected, evenly spaced out across the back. In each 1-mm area, the thickness of the epidermis was measured by drawing lines in Adobe Illustrator that span the width of the epidermal layer, then using the 200-μm scale in each image to convert line length to microns. Five measurements were made that evenly spanned the 1-mm area; each measurement was made at the widest local area.

## Data and code availability

The published article includes all datasets and code generated or analyzed during this study.

## Supplemental information

Document S1. Figures S1–S4 and Tables S1–S3

Document S2. Article plus supplemental information
