# Supplementary material for: A model organism pipeline provides insight into the clinical heterogeneity of TARS1 loss-of-function variants
Source: HGG Adv. 2024 Jul 2;5(3):100324. doi: 10.1016/j.xhgg.2024.100324 (PMC11284558; doi:10.1016/j.xhgg.2024.100324)
Supplement: Document S1. Figures S1–S4 and Tables S1–S3 [file mmc1.pdf]

Human Genetics and Genomics Advances, Volume *5*

Supplemental information

A model organism pipeline provides insight into the clinical heterogeneity of *TARS1* loss-of-function variants

Rebecca Meyer-Schuman, Allison R. Cale, Jennifer A. Pierluissi, Kira E. Jonatzke, Young N. Park, Guy M. Lenk, Stephanie N. Oprescu, Marina A. Grachtchouk, Andrzej A. Dlugosz, Asim A. Beg, Miriam H. Meisler, and Anthony Antonellis

Supplemental Materials:

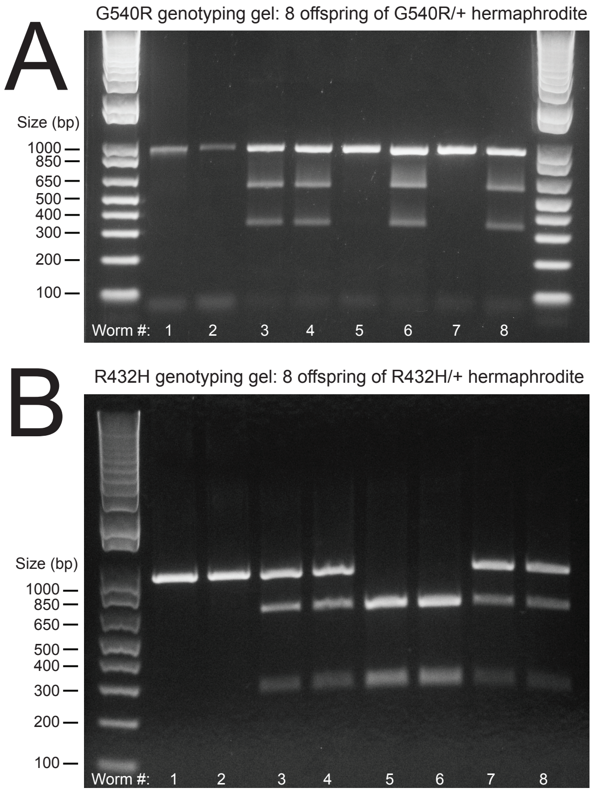

Figure S1. **Genotyping G540R and R432H *tars-1 C. elegans* lines. (A)** Genotyping of 8 offspring from a G540R/+ hermaphrodite. The wild-type amplicon is 878bp, but in the presence of the G540R allele, *Eag*I digests this amplicon into 550bp and 328bp fragments. Worms 1, 2, 5, and 7 are *tars-1* +/+; worms 3, 4, 6, and 8 are *tars-1* G540R/+. **(B)** Genotyping of 8 offspring from a R432H/+ hermaphrodite. In the presence of the R432H allele, *Sac*I digests the 884bp amplicon into 617bp and 267bp fragments. Worms 1 and 2 are *tars-1* +/+; worms 3, 4, 7, and 8 are R432H/+; worms 5 and 6 are R432H/R432H.

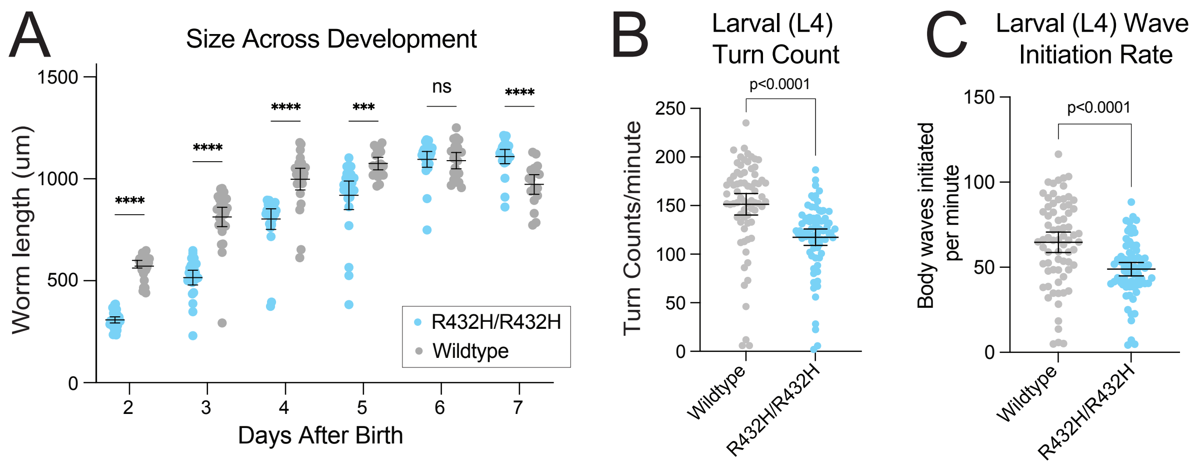

Figure S2. **R432H impairs locomotion at the L4 larval stage. (A)** Body length measurements of R432H/R432H *tars-1* worms and wild-type *tars-1* worms from two to six days after birth. For R432H/R432H, 24-29 worms were measured each day. For wild-type worms, 19-35 were measured each day. **(B)** Turn counts per minute for R432H/R432H worms (n=73) and wild-type worms (n=71) at larval stage L4, which was identified based on gonadal development. **(C)** Rate of wave initiations from either the head or the tail for L4 R432H/R432H worms (n=73) and wild-type worms (n=71). For all panels, bars indicate mean value and 95% confidence intervals. Statistical significance was evaluated using an unpaired t-test with Welch's correction; ****, $p<0.0001$; ***, $p<0.001$ ns=not significant.

A

Offspring of R432H/+ x R432H/+

	+/+	R432H/+	R432H/R432H
Observed	13	24	6
Expected	10.75	21.5	10.75

p=0.2393

B

Offspring of F538Kfs*4/+ x F538Kfs*4/+

	+/+	F538Kfs*4/+	F538Kfs*4/F538Kfs*4
Observed	7	21	0
Expected	7	14	7

p=0.0052

C

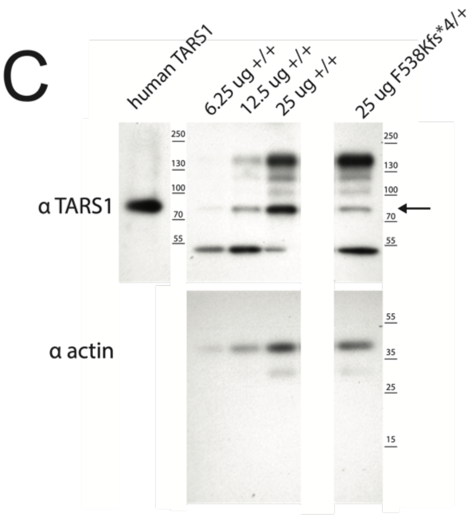

Figure S3. **Viability of R432H or F538Kfs*4 homozygous mice.** **(A)** Genotype analysis of 28 offspring from *Tars1*$^{F538Kfs*4/+}$ x *Tars1*$^{F538Kfs*4/+}$ mouse mating pairs. **(B)** Genotype analysis of 43 offspring from *Tars1*$^{R432H/+}$ x *Tars1*$^{R432H/+}$ mouse mating pairs. All mice were genotyped at approximately 3 weeks of age. Chi-square tests were performed to determine if the difference between observed genotype counts and expected genotype counts was statistically significant. **(C)** Representative western blot image for Tars1 protein in brain tissue of wild-type *Tars1* mice and *Tars1*$^{F538Kfs*4/+}$ mice. Human TARS1 (predicted size of 83 kDa), expressed in yeast, is shown on the left as a size control. An arrow points to the band corresponding to Tars1 (predicted size of 83 kDa). For wild-type mice, 6.25µg, 12.5µg, and 25µg lysate was loaded as a comparison to 25µg lysate for *Tars1*$^{F538Kfs*4/+}$ samples.

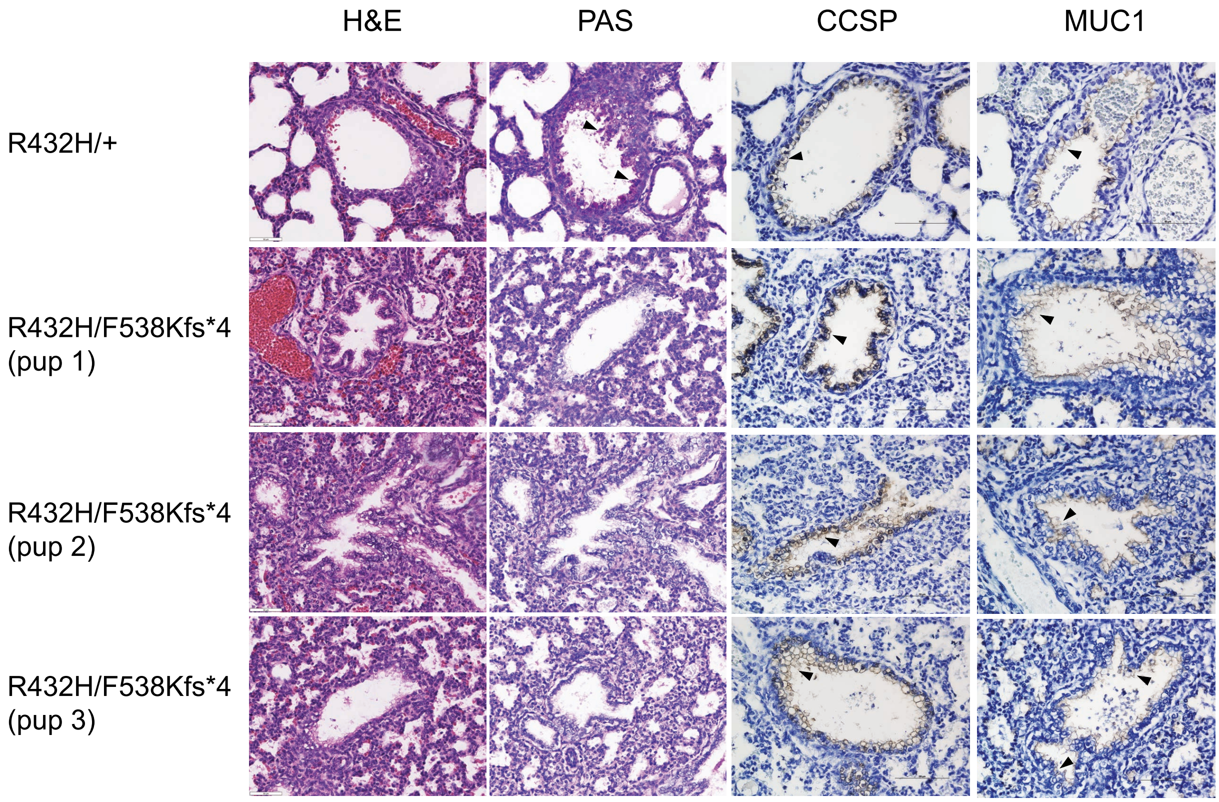

Figure S4. Club cell secretory protein and mucin 1 are present in bronchioles of R432H/F538Kfs*4 mice. Lung sections stained with (from left to right) H&E, PAS, CCSP antibody, or MUC1 antibody. The R432H/+ images (top row) are representative of three R432H/+ P0 pups, and the R432H/F538Kfs*4 images (bottom three rows) are representative of five R432H/F538Kfs*4 P0 pups. Black arrows point to the PAS signal in the R432H/+ mouse, and the CCSP and MUC1 signals in all mice. The CCSP and MUC1 signals are shown in brown, with the hematoxylin counterstain in dark blue.

***TARS1* variant (*H. sapiens*)**	***tars-1* variant (*C. elegans*)**	***Tars1* variant (*M. musculus*)**
R433H	R432H	R432H
G541R	G540R	

Table S1. Comparison of orthologous amino-acid codons between human *TARS1*, worm *tars-1*, and mouse *Tars1*.

Primer name	Sequence
TARS1 ORF GW F	GGGGACAAGTTTGTACAAAAAAGCAGGCTCAATG TTTGAGGAGAAGGCCAGC
TARS1 ORF GW R	GGGGACCACTTTGTACAAGAAAGCTGGGTTTTAAA ATTCTTCTTCTGC
G541R *TARS1* Mut F	GATGGAGCTTTCTATCGCCCAAAGATTGACATAC
G541R *TARS1* Mut R	GTATGTCAATCTTTGGGCGATAGAAAGCTCCATC
R433H *TARS1* Mut F	CGAGAACTGCCTCTGCACCTAGCTGATTTTGGG
R433H *TARS1* Mut R	CCCAAAATCAGCTAGGTGCAGAGGCAGTTCTCG
N412Y *TARS1* Mut F	CCTGAAACCCATGTACTGCCCAGGACACTGC
N412Y *TARS1* Mut R	GCAGTGTCCTGGGCAGTACATGGGTTTCAGG
G540R *tars-1* genotyping F	CGTCAAGATCAAATTTCCGAGG
G540R *tars-1* genotyping alternate F	GGGGCATTGTCTCATGTTCG
G540R *tars-1* genotyping R	TGCATAGTCTCTGACTGACTCG
R432H *tars-1* genotyping F	ATCGGCGTTCTGGTATCC
R432H *tars-1* genotyping R	AAGCGTTGAGCGCATTGG
F538Kfs*4 *Tars1* genotyping F	GTGTCGAGTGAGGGAATGTTTTCTGTA
F538Kfs*4 *Tars1* genotyping R	TTAAACCTGATGGGCAACTGAAA
R432H *Tars1* genotyping F	CTTCTATTGCTCTGTGAGGC
R432H *Tars1* genotyping R	TATCTCCCTTCACCTGTAACC

Table S2. **Sequences for primers used in this study.** All primers are listed 5’ to 3’.

**Oligonucleotide**	**Sequence**
G540R *tars-1* sgRNA	CCGGGAGATGGAGCATTCTACGG
R432H *tars-1* sgRNA	CCATTCCGATTCGCTGATTTTGG
G540R *tars-1* ssODN repair template	TCAGGCAGAAAA TGGGTGTTGAACCCGGGAGA TGGAGCATTCTACCGGCCGAAAATCGATATCACCATTCAAG AT GCTCTCAAGAGAAA
R432H *tars-1* ssODN repair template	GTCTCATGTTCGGACACATGCCACACACCTACAATGAGCTC CCGTTTCA TTTTGCAGACTTTGGAGTTTTGCACAGAAA TGAAA TGTCTGGTGCTTTGACTG
F538Kfs*4 *tars-1* sgRNA	CTGGAGATGGAGCCTTCTAT
R432H *Tars1* sgRNA	ACCAAAGTCAGCTAGCCGCA
R432H *Tars1* ssODN repair template	CCTTGCTTTCTCCTTCAGCCTGATGTTCGATCACCGGCCACG GTCCTGGCGAGAGCTGCCACTGCACCTGGCAGACTTTGGTG TGCTGCATAGGAATGAGCTCTCGGGGGCTC

Table S3. **Sequences for sgRNAs and ssODNs used in this study.** All sequences are listed 5’ to 3’.

Supplemental Methods

*Generation of the F538Kfs*4 Tars1 mouse line*

The F538Kfs*4 allele was generated similar to the R432H allele, using a sgRNA that targeted exon 14. This generated an 11 base pair deletion that ablates a *Hae*III cut site and lead to a premature stop codon shortly downstream of the PAM site (F538Kfs*4). To genotype this deletion, the region was amplified (primer sequences in Table S2) and digested with *Hae*III; an undigested upper band indicated the presence of the frameshift allele. To evaluate the Mendelian ratios of offspring genotypes, 28 pups from crosses between *Tars1*$^{F538Kfs*4/+}$ females and *Tars1*$^{F538Kfs*4/+}$ males were genotyped using this restriction enzyme digest strategy.

Western blot analyses from mouse brain

Total protein concentration was measured using the Thermo Scientific Pierce BCA Protein Assay Kit. 6.25μg, 12.5μg, or 25μg of lysate was analyzed. Samples were prepared with 1X Novex Tris-Glycine SDS sample buffer (Invitrogen) and 2-mercaptoethanol (BME), and were boiled at 99°C for 5 minutes. Protein samples were separated on precast 4-20% Novex Wedgewell Tris-glycine gels (Invitrogen) at 150V for 1 hour and 15 minutes. PVDF membranes (Millipore Sigma) were pre-washed in 100% methanol for 1 minute, then soaked in 1X transfer buffer (Invitrogen) and 10% methanol between two pieces of filter paper (Thermo Fisher Scientific). Samples were transferred from the Tris-Glycine gel to the PVDF membrane using a Mini Trans-Blot Electrophoretic Transfer Cell (Biorad) at 0.03A for 18-20 hours. The membrane was blocked in a 2% solution of non-fat dry milk powder in 1X TBST overnight at 4°C. After blocking, the membrane was washed with 1X TBST three times, with each wash comprising five minutes of rocking at room temperature. Primary antibody was applied in a 2% milk solution: anti-TARS1 (Thermo Fisher PA5-30690) was applied at 1:500 dilution and anti-actin (Sigma

A5060) was applied as a loading control at 1:5,000 dilution. Primary antibody was incubated overnight at 4°C. Membranes were washed three times with 1X TBST as above, and secondary antibodies (anti-mouse HRP [1:2,000; Thermo Fisher Scientific] for TARS1 and anti-rabbit HRP [1:5,000; EMD Millipore] for actin) were applied in 2% milk solution. The blots were rocked for 1 hour at room temperature before incubating with SuperSignal West Dura substrate (Thermo Scientific) according to the manufacturer's instructions.

Immunohistochemistry

Paraffin sections were deparaffinized and rehydrated through a graded ethanol series. Endogenous peroxidase activity was quenched by treatment with Peroxidase Suppressor Reagent for 15 minutes (Pierce™ Peroxidase IHC Detection Kit, Thermo Scientific, 36000). Heat induced epitope retrieval was performed by incubating slides in antigen retrieval agent (R&D Systems, CTS015) at 95°C for 5 minutes and then allowing the slides to cool at room temperature for 10 minutes. Slides were blocked in Universal Blocker Blocking Buffer in TBS (Pierce™ Peroxidase IHC Detection Kit, Thermo Scientific, 36000) for one hour. Primary antibodies (see below) were diluted in blocking buffer and slides were incubated with primary antibody overnight at 4°C. After washing with PBS, slides were incubated in secondary antibody for one hour at room temperature. Substrate incubation, chromogenic development, and hematoxylin counterstain were performed according to manufacturer recommendation (Pierce™ Peroxidase IHC Detection Kit, Thermo Scientific, 36000). Primary antibodies used were rabbit anti-CCSP (Sigma-Aldrich, 07-623) 1:1000 and rabbit anti-MUC1 (Abcam, ab15481) 1:100. For anti-MUC1 staining, secondary antibody (Sigma Aldrich, 12-348) was used at a dilution of 1:500; for anti-CCSP staining, secondary antibody was used at a dilution of 1:1000.
